# The Potential of α-Spinasterol to Mimic the Membrane Properties of Natural Cholesterol

**DOI:** 10.3390/molecules22081390

**Published:** 2017-08-22

**Authors:** Ivan Haralampiev, Holger A. Scheidt, Daniel Huster, Peter Müller

**Affiliations:** 1 Department of Biology, Humboldt-Universität zu Berlin, Invalidenstr. 42, D-10115 Berlin, Germany; ivans.haralampiev@gmail.com; 2 Institute for Medical Physics and Biophysics, Leipzig University, Härtelstr. 16–18, D-04107 Leipzig, Germany; holger.scheidt@medizin.uni-leipzig.de (H.A.S.); daniel.huster@medizin.uni-leipzig.de (D.H.)

**Keywords:** cholesterol, α-spinasterol, membrane structure, membrane permeability, lateral domains, NMR, fluorescence

## Abstract

Sterols play a unique role for the structural and dynamical organization of membranes. The current study reports data on the membrane properties of the phytosterol (3β,5α,22*E*)-stigmasta-7,22-dien-3-β-ol (α-spinasterol), which represents an important component of argan oil and have not been investigated so far in molecular detail. In particular, the impact of α-spinasterol on the structure and organization of lipid membranes was investigated and compared with those of cholesterol. Various membrane parameters such as the molecular packing of the phospholipid fatty acyl chains, the membrane permeability toward polar molecules, and the formation of lateral membrane domains were studied. The experiments were performed on lipid vesicles using methods of NMR spectroscopy and fluorescence spectroscopy and microscopy. The results show that α-spinasterol resembles the membrane behavior of cholesterol to some degree.

## 1. Introduction

The structural organization of cellular membranes is determined by a number of physico-chemical interactions between the individual membrane components, which allow membranes to realize the unique functions they play in complex cellular homeostasis. Of these interactions, the interplay between cholesterol and the other membrane constituents (phospholipids and membrane proteins) is of special relevance in mammals, as cholesterol, due to its unique structure, is able to significantly modify important membrane properties such as dynamic formation of lateral membrane domains, also called rafts [1,2,3,4], molecular packing of the acyl chains of phospholipids [5], and membrane permeability [6,7]. While in mammalians, cholesterol is the molecule playing the decisive role for bringing about these specific membrane properties, in fungi and plants, other sterols such as ergosterol, campesterol, sitosterol, stigmasterol, and (3β,5α,22*E*)-stigmasta-7,22-dien-3-β-ol (α-spinasterol) take over the role of cholesterol. These sterols, which are structurally somewhat similar to cholesterol, have received special attention because of their putatively positive impact on human health. To understand this influence in molecular detail, several studies have investigated their cellular and pharmacological activities e.g., for preventing and treating the impacts of physiological dysregulations like in cardiovascular and neurodegenerative diseases [8].

**Figure 1:**
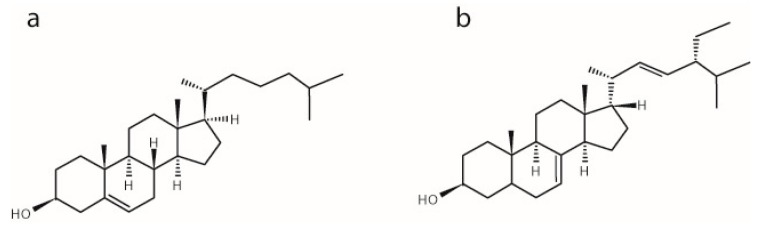
Chemical structures of (**a**) cholesterol and (**b**) α-spinasterol.

α-Spinasterol, which is found in argan oil (from *Argania spinose*) and in seed oil (from cactus pear, *Opuntia ficus indica*), is a component of natural food ingredients. e.g., argan oil is used as a traditional food ingredient in the ‘Amazigh diet’ and provides about 25% of the total diet fat intake to the indigenous consumers [9]. Several studies have underlined the health benefits of this oil, e.g., its reducing capacity for the plasma LDL-cholesterol level [10]. In searching for the components responsible for these positive effects, α-spinasterol was proposed to be one of the main mediator molecules. This phytosterol differs from cholesterol by (i) an additional ethyl group and double bond in the aliphatic side chain; and (ii) the position of the double bond in the tetracyclic ring system (Figure 1). Investigating the physiological effect(s) of α-spinasterol, several studies showed that it modulates mitochondrial activity and gene expression of nuclear receptors [11,12], exhibits antitumor, antioxidative, and anti-inflammatory activities [13,14], and influences serum concentrations and metabolism of cholesterol in rats [15].

To understand the physiological role of α-spinasterol in more detail, its influence on membrane properties is of specific interest. However, to the best of our knowledge, the membrane behavior of α-spinasterol, especially in comparison with cholesterol has not been investigated so far. Therefore, we characterized the influence of α-spinasterol on important membrane parameters and compared its membrane behavior to that of cholesterol. To this end, the influence of α-spinasterol on (i) the molecular packing of membrane lipids; (ii) the membrane permeability of lipid bilayers; and (iii) its ability to support lateral membrane domains was investigated by using methods of NMR spectroscopy and fluorescence spectroscopy and microscopy. Our data reveal that α-spinasterol shows membrane properties, which are similar to those of cholesterol, but the impact for some of the investigated parameters is different.

## 2. Results

### 2.1. The Influence of α-Spinasterol on Lipid Chain Packing

**Figure 2:**
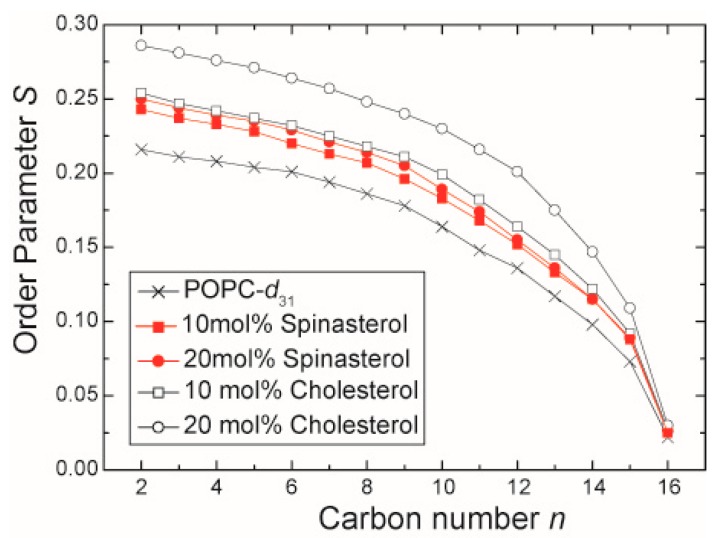
^2^H-NMR chain order parameter of the *sn*-1 chain of POPC-*d*_31_ in the presence of 10 and 20 mol % α-spinasterol. For comparison, the chain order parameters of pure POPC-*d*_31_ and POPC-*d*_31_ membranes in the presence of 10 and 20 mol % cholesterol are shown (data adopted from [18]). The error of measurement is smaller than the symbol size.

To investigate the effect of α-spinasterol on lipid chain packing and its capability to induce lipid condensation, static ^2^H-NMR measurements using chain deuterated 1-palmitoyl-*d*_31_-2-oleoyl-*sn*-glycero-3-phosphocholine (POPC-*d*_31_) were performed. Lipid condensation describes the preferential interaction of cholesterol with saturated lipid chains that leads to an increase in phospholipid chain length and a decrease in the cross-sectional area per molecule in the membrane. The obtained NMR spectra of the membranes in the presence of 10 and 20 mol % α-spinasterol exhibit the typical NMR powder line shape of lamellar liquid-crystalline lipid membranes (spectra not shown). The NMR spectra were deconvoluted by dePakeing, and order parameters were determined as described in the literature [16,17]. The lipid chain order parameters from POPC membranes in the absence and in the presence of α-spinasterol are shown in Figure 2. For comparison, the order parameters of POPC-*d*_31_ in the presence of 10 and 20 mol % cholesterol are also shown (from [18]). Since ^2^H-NMR order parameters represent a very reliable and highly reproducible way of obtaining information about the lipid chain dynamics, please note that the error of measurements in these plots is smaller than symbol size. α-Spinasterol clearly fails to induce the same lipid condensation effect as the same amount of cholesterol does. The chain order parameters in the presence of 20 mol % α-spinasterol are comparable to the effect of only 10 mol % cholesterol. This difference is also reflected in the lipid chain length, which was calculated for the individual samples (Table 1). Since the condensation effect of 10 mol % α-spinasterol in the membrane is not simply half of that for 20 mol %, a saturation behavior has to be assumed as it was observed for other sterols (but not for cholesterol) [19].

### 2.2. Influence of α-Spinasterol on Membrane Permeability

**Figure 3:**
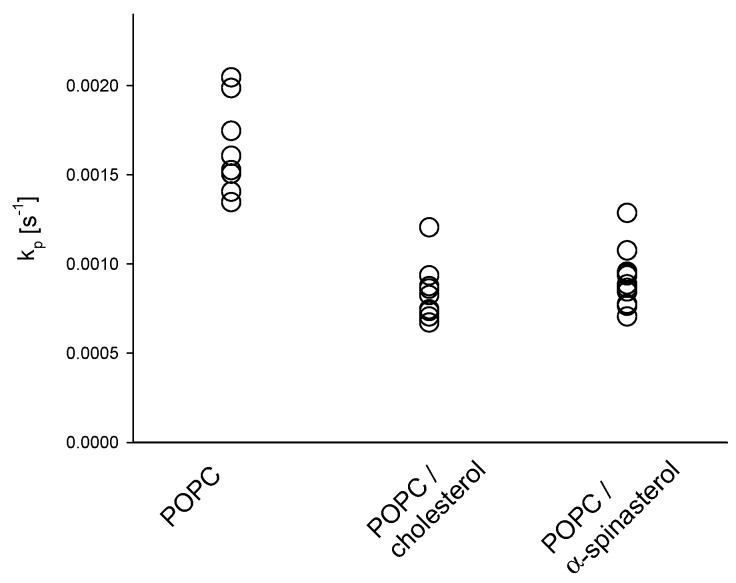
Rate constants (k_p_) for the permeation of dithionite across large unilamellar vesicle (LUV) membranes composed of POPC in the absence and in the presence of 20 mol % cholesterol or α-spinasterol at 37 °C. All single values of the rate constant are shown which were determined from two independent samples each threefold measured.

The influence of α-spinasterol on membrane permeability was determined using a fluorescence assay, which measures the permeation of dithionite ions across lipid membranes [18,22]. The rate constants for dithionite permeation of 1-palmitoyl-2-oleoyl-*sn*-glycero-3-phosphocholine (POPC) large unilamellar vesicles (LUVs) in the absence or in the presence of either cholesterol or α-spinasterol are shown in Figure 3. A comparison of pure lipid vesicles in the absence and in the presence of cholesterol reveals that this sterol caused the well-known decrease of permeability toward polar molecules [23]. The rate constants in the presence of α-spinasterol were similar to those of cholesterol underlining that the phytosterol has a comparable impact on reducing membrane permeability.

### 2.3. Influence of α-Spinasterol on the Formation of Lateral Domains in Giant Unilamellar Vesicles (GUVs)

**Figure 4:**
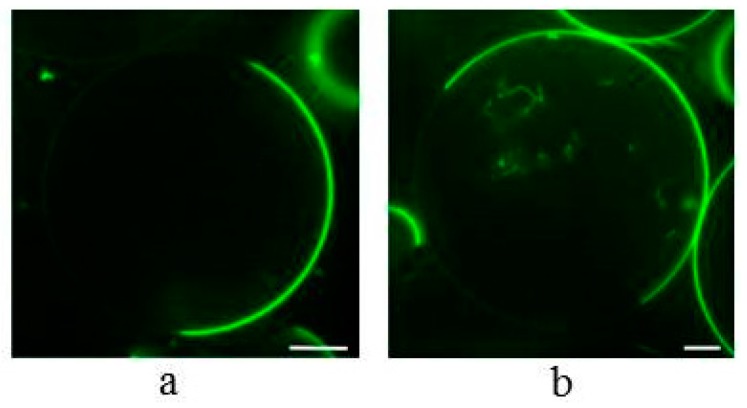
Confocal fluorescence images of Giant Unilamellar Vesicles (GUVs) containing DOPC/SSM/cholesterol (1:1:1) (**a**) or DOPC/SSM/α-spinasterol (1:1:1) (**b**). The GUV membranes were labeled with *N*-Rh-DOPE (0.5 mol %) that sorts preferentially into liquid disordered (ld) domains. Bar corresponds to 10 µm.

We continued to investigate whether α-spinasterol is also able to induce the formation of lateral membrane domains as cholesterol does. It has been well established that cholesterol triggers the formation of liquid disordered (ld) and ordered (lo) domains e.g., in GUVs or multilamellar vesicles (MLVs) consisting of equimolar amounts of phospholipid, cholesterol, and sphingomyelin, and representing the canonical lipid raft mix [4,24,25,26,27,28]. In line with this, we observed lateral domains in 1,2-dioeloyl-*sn*-glycero-3-phosphocholine (DOPC)/sphingosylphosphorylcholine (SSM)/cholesterol GUVs (Figure 4a). The domain structure was visualized by additionally labeling the membrane with the ld marker 1,2-dioleoyl-*sn*-glycero-3-phosphoethanolamine-*N*-(lissamine rhodamine B sulfonyl) (ammonium salt) (*N*-Rh-DOPE). The fluorescence microscopic images show membrane regions of low and of high fluorescence intensity, representing the lo and ld phases, respectively. When cholesterol was substituted by α-spinasterol, the vesicles show a similar lateral domain pattern (Figure 4b).

**Figure 5:**
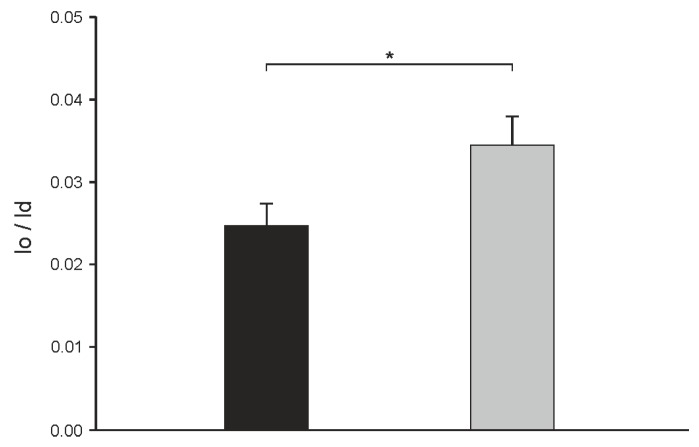
Distribution of the lipid analog *N*-Rh-DOPE (0.5 mol %) between the lo and the ld domain in GUVs consisting of DOPC/SSM/cholesterol (1:1:1) (black column) or DOPC/SSM/α-spinasterol (1:1:1) (gray column). From images as shown in Figure 4, fluorescence intensities of *N*-Rh-DOPE localized in lo and ld domains were determined and a lo/ld ratio was calculated (see Materials and Methods). The data represent the mean ± SE of 60 vesicles, *p*-value < 3% (corresponding to *).

In order to compare the distribution of *N*-Rh-DOPE in both domains between cholesterol- and α-spinasterol-containing vesicles quantitatively, the fluorescence intensities of membrane areas localized in lo and ld domains were determined calculating a lo/ld ratio (see Materials and Methods). To avoid the results to be influenced by slight variations of the lipid composition of different vesicles [4], a total of 60 single GUVs of each lipid mixture were analyzed. Figure 5 shows that *N*-Rh-DOPE mainly accumulates in the disordered domain for both vesicle species as seen from the very low lo/ld values. However, for α-spinasterol a larger lo/ld ratio was measured indicating that this sterol is not able to fully mimic cholesterol in this regard.

## 3. Discussion

In the current study, we investigated by how much the plant sterol α-spinasterol reproduces the very unique membrane properties of cholesterol in lipid membranes by comparing the influence of both sterols on lipid chain order, membrane permeability, and formation of lateral membrane domains. Comparing the membrane properties of cholesterol with those of other sterols supports the molecular understanding of the unique impact of cholesterol on membranes and its physiological roles. Such molecules comprise precursors in the cholesterol synthesis, naturally occurring sterols, and artificial fluorescence and EPR analogues of cholesterol [19,29,30,31,32,33,34,35]. Previous biophysical investigations indicated that already small alterations in the structure of the sterol molecule cause significant differences in the membrane properties of the respective molecule [2,6,36,37,38,39].

The data reported in this study also support the unique structure-function relationship of native cholesterol and reveal differences of α-spinasterol with regard to membrane effects. First, α-spinasterol, like cholesterol, condenses the fatty acyl chains of phospholipids by increasing their degree of order. However, comparing the data on a quantitative level, the phytosterol α-spinasterol was less effective than the mammalian sterol cholesterol: about twice the α-spinasterol concentration was necessary to approximately achieve the same lipid chain condensation as induced by cholesterol. α-Spinasterol features several structural differences compared to cholesterol. With regard to these features, we hypothesize that the additional ethyl group and double bond localized in the aliphatic side chain of the molecule mainly cause the decreased ordering effect of the sterol. Those structural modifications decrease the ability of α-spinasterol to intercalate among and to order the POPC acyl chains and possibly represent defects leading to a less dense packing of the molecules in the membrane. Another phytosterol, stigmasterol, having the same modifications in the alkyl side chain like α-spinasterol but the double bond in the ring system at the same position as cholesterol, can also not fully mimic cholesterol with regard to membrane ordering [19]. Notably, this impact was concentration dependent in that stigmasterol caused similar membrane ordering like cholesterol up to a concentrations of about 10 mol %, but had a decreased influence at higher concentration [19]. Here, we observed for α-spinasterol a smaller influence compared to cholesterol also at 10 mol %. These differences might be explained by the different position of the single double bond in the sterol tetracyclic ring, which is the same for stigmasterol and cholesterol, but different for α-spinasterol. Those modifications may influence the van der Waals interactions between lipid chains and the respective sterol. Nevertheless, with regard to the amount of cholesterol in plasma membranes, mainly the larger sterol concentrations are physiologically relevant.

Second, despite the reduced ability of α-spinasterol to affect the ordering of fatty acyl chains, the phytosterol resembles endogenous cholesterol in decreasing the permeability of POPC membranes toward the polar molecule dithionite. Interestingly, Schuler and coworkers found that stigmasterol causes not only a low efficiency in lipid ordering, but also a decreased water permeability across lipid membranes [29]. However, in this study, the authors (i) used membranes consisting of soybean PC; (ii) determined water permeation indirectly by measuring osmotic swelling of vesicles; and (iii) investigated only sterol concentrations up to 15 mol %.

Third, α-spinasterol triggered the formation of lateral domains in GUV membranes. Comparing the distribution of a fluorescence marker between these domains, the extent of the distribution was slightly lower for α-spinasterol compared to cholesterol. The presence and physiological relevance of lateral membrane domains in biological membranes, especially plasma membranes, have been investigated since the 80’s. For that, numerous approaches have been applied using model membranes as well as manipulated and untreated biological membranes [1,40,41,42,43]. Whereas, with regard to the latter, the first studies were mainly performed on mammalian cells, meanwhile the presence of those domains have also been proposed for plant cells [44,45,46]. This indicates that phytosterols are also able to realize the unique interactions with certain phospholipids in plant cells leading to the formation of segregated membrane components. The order of these domains and their stability depends on the structure of the respective partitioning sterol [47,48,49,50,51]. Interestingly, to the best of our knowledge those studies have not used the GUV assay in which the lateral distribution of fluorescent lipids is determined by fluorescence microscopy, an approach which has been widely applied for cholesterol and related sterols. Using GUVs, it is shown here that α-spinasterol mimics cholesterol in triggering phase separation in the trinary lipid mixture DOPC, SSM, and sterol. This role of α-spinasterol for domain formation is supported by another study finding an enrichment of this sterol in Triton X-100-insoluble membranes isolated from plasma membranes of *Medicago truncatula* roots [52]. Several studies proposed for the structurally related sterol stigmasterol (see above) a lower efficiency to promote the formation of liquid-ordered domains compared to cholesterol [47,49,51]. From this, it can be concluded that the different position of the double bond in the sterol ring of α-spinasterol and stigmasterol plays the crucial role for explaining their different lateral distribution. It should be mentioned that our data focus on the canonical equimolar raft mixture of DOPC/SSM/cholesterol [4]. It is conceivable that other combinations of the three lipids may produce an altered domain formation as domain formation is triggered by the interactions between the individual lipids, the probability for such stabilizing or destabilizing interactions to occur of course varies within the ternary phase diagram.

Summarizing the data underlines that α-spinasterol is able to modulate important membrane properties similar to cholesterol, however, to a different extent for some of these parameters. Our study may contribute to understand the basis for the positive effects of α-spinasterol when used as food ingredient or medical therapeutics. The results show that on the structural level of lipid membranes, this sterol is able to partially take over the role of natural cholesterol. However, the importance of cholesterol for physiological processes is much more complex. Therefore, more experiments also covering higher organization levels of life are required.

## 4. Materials and Methods

### 4.1. Materials

All lipids, POPC, DOPC, SSM, POPC-*d*_31_, 1-palmitoyl-2-(12-[*N*-(7-nitrobenz-2-oxa-1,3-diazol-4-yl)amino]dodecanoyl]-*sn*-glycero-3-phosphocholine (NBD-PC), *N*-Rh-DOPE, and cholesterol were purchased from Avanti Polar Lipids, Inc. (Alabaster, AL, USA). α-Spinasterol was from Tocris Biosciences (Bristol, UK). All other chemicals were purchased from Sigma-Aldrich (Taufkirchen, Germany) and were used without further purification.

### 4.2. Preparation of NMR Samples

α-Spinasterol and POPC-*d*_31_ were dissolved in chloroform at a molar ratio of 1:4 (mol/mol). After evaporation of the solvent, the samples were re-dissolved in cyclohexane and lyophilized overnight at high vacuum. The obtained fluffy powder was hydrated with 40 wt. % deuterium-depleted water; the samples were equilibrated by several freeze-thaw cycles and gentle centrifugation and finally transferred into 5 mm glass vials.

### 4.3. ^2^H-NMR Experiments

The ^2^H-NMR experiments were performed on a Bruker DRX300 NMR spectrometer (Bruker BioSpin, Rheinstetten, Germany) at a resonance frequency of 46.1 MHz for ^2^H using a solids probe with a 5-mm solenoid coil. ^2^H-NMR spectra were acquired using a quadrupolar echo pulse sequence [53] with a relaxation delay of 1 s. The two π/2 pulses with a typical length of around 3.2 µs were separated by a 50 µs delay. The spectral width was 500 kHz. ^2^H-NMR spectra were dePaked and smoothed order parameters were determined as described previously [16]. From these order parameters the lipid chain extent as a measure of the lipid chain length was calculated according to the mean torque model [20,21].

### 4.4. Preparation of LUVs

LUVs were prepared by the extrusion method [54]. Aliquots of lipids were dissolved in chloroform and dried in a rotating round-bottom flask under vacuum until a lipid film was formed. Lipids were resuspended at first in a small volume of ethanol (final ethanol concentration was below 1% (*v/v*)). Subsequently, HBS (HEPES buffered saline, 145 mM NaCl and 10 mM HEPES, pH 7.4) was added to reach a final lipid concentration of 1 mM and the mixture was vortexed. To prepare LUVs, this suspension was subjected to five freeze-thaw cycles followed by extrusion of the lipid suspension 10 times through 0.1 μm polycarbonate filters at 40 °C (extruder from Lipex Biomembranes Inc., Vancouver, BC, Canada; filters from Costar, Nucleopore, Tübingen, Germany).

### 4.5. Preparation of GUVs

GUVs were prepared using the electro swelling method [55]. Lipid mixtures were prepared from stock solutions in chloroform. Finally, 100 nmol of the domain forming lipid mixture of DOPC, SSM, and cholesterol or α-spinasterol (1:1:1, molar ratio) including 0.5 mol % of the ld domain marker *N*-Rh-DOPE were dissolved in chloroform and spotted onto custom-built titan chambers. These were placed on a heater plate at 50 °C to facilitate solvent evaporation, and subsequently put under high vacuum for at least 1 h for evaporation of remaining traces of solvent. Lipid-coated slides were assembled using a spacer of Parafilm (Pechiney Plastic Packaging, Chicago, IL, USA) for insulation. The electro swelling chamber was filled with 1 ml sucrose buffer (250 mM sucrose, 15 mM NaN_3_, osmolarity of 280 mOsm/kg) and sealed with plasticine. An alternating electrical field of 10 Hz rising from 0.02 V to 1.1 V in the first 56 min was applied for 2.5 h at 55 °C.

### 4.6. Permeation Assay

The permeation of dithionite ions across lipid membranes was measured as described [18,22]. LUVs containing POPC and 0.5 mol % NBD-PC without and with 20 mol % of cholesterol or α-spinasterol were prepared. The NBD fluorescence intensity of 33 μM LUVs was recorded in a cuvette at 540 nm (λ_ex_ = 470 nm, slit width for excitation and emission 4 nm) at 37 °C using an Aminco Bowman Series 2 spectrofluorometer. After 30 s, sodium dithionite was added from a 1 M stock solution in 100 mM Tris (pH 10.0) to give a final concentration of 50 mM. Dithionite ions rapidly quench the fluorescence of analog molecules localized in the outer leaflet, which is reflected by a rapid initial decrease of fluorescence intensity (kinetics not shown). Subsequently, the fluorescence intensity decreased slowly caused by a slow permeation of dithionite ions across the bilayer. By that process, dithionite reacted with the NBD-PC molecules in the inner leaflet. After 300 s, Triton X-100 (0.5% (*w/v*) final concentration) was added, enabling complete reaction of dithionite with NBD-PC, resulting in a complete loss of fluorescence. The curves were normalized to the fluorescence intensities before addition of dithionite and were fitted to a bi-exponential equation. From the fittings, the rate constants for the rapid fluorescence decrease (representing reduction of NBD-PC in the outer leaflet) and those for the slow decrease (representing permeation of dithionite across the bilayer) were determined. The latter ones were used as the parameter for membrane permeability. Note, that the slow fluorescence decay could also be explained by a transbilayer movement of NBD-PC from the inner to the outer leaflet (flip-flop). However, the transbilayer movement of phospholipids in lipid bilayers is very slow, with half times in the time scale of hours.

### 4.7. Confocal Laser Scanning Microscopy

For microscopy, a Visitron VisiScope scanning disk confocal laser microscope (Visitron Systems, Puchheim, Germany) with a 60× oil objective and an Andor iXon 888 EMCCD camera (1024 × 1024 pixels, Andor, Belfast, UK) were used. *N*-Rh-DOPE was excited by a 561 nm diode laser. GUVs were mixed 1:1 with 250 mM glucose buffer (5.8 mM NaH_2_PO_4_, 5.8 mM Na_2_HPO_4_, osmolarity of 300 mOsm/kg, pH 7.2) in poly-lysine coated glass bottom culture dishes (MatTek Corporation, Ashland, MA, USA). Vesicles were allowed to settle down some minutes before acquisition of z-stacks with 1 µm step size.

### 4.8. Image Analysis

For image analysis, the equatorial plane of a vesicle was used. Distribution of *N*-Rh-DOPE across the ld and lo domain was measured as described in [56]. Four regions of interest (ROI, squares) with equal areas were positioned on each, the ld and the lo domain (membrane ROIs). For all positions, another squared ROI near the membrane ROIs were used to determine background signal. The mean fluorescence intensity of each membrane ROI was calculated and corrected by subtraction of the corresponding mean background ROI intensity. The mean value of all lo regions of a single GUV was divided by the mean value all ld regions.

## Figures and Tables

**Table 1:** Lipid chain length calculated according to the mean torque model [20,21].

Sample	Chain Length/Å
10 mol % α-spinasterol/POPC-*d*_31_	12.2
20 mol % α-spinasterol/POPC-*d*_31_	12.4
pure POPC-*d*_31_	11.7
10 mol % cholesterol/POPC-*d*_31_	12.5
20 mol % cholesterol/POPC-*d*_31_	13.2
